# Aluminum surface defect detection method based on a lightweight YOLOv4 network

**DOI:** 10.1038/s41598-023-38085-x

**Published:** 2023-07-08

**Authors:** Songsong Li, Shangrong Guo, Zhaolong Han, Chen Kou, Benchi Huang, Minghui Luan

**Affiliations:** College of Information Engineering, Dalian Ocean University, Dalian, 116021 China

**Keywords:** Machine learning, Data processing, Network models

## Abstract

Deep learning is currently being used to automate surface defect detection in aluminum. The common target detection models based on neural networks often have a large number of parameters and a slow detection speed, which is not conducive to real-time detection. Therefore, this paper proposes a lightweight aluminum surface defect detection model, M2-BL-YOLOv4, based on the YOLOv4 algorithm. First, in the YOLOv4 model, the complex CSPDarkNet53 backbone network was modified into an inverted residual structure, which greatly reduced the number of parameters in the model and increased the detection speed. Second, a new feature fusion network, BiFPN-Lite, is designed to improve the fusion ability of the network and further improve its detection accuracy. The final results show that the mean average precision of the improved lightweight YOLOv4 algorithm in the aluminum surface defect test set reaches 93.5%, the number of model parameters is reduced to 60% of the original, and the number of frames per second (FPS) detected is 52.99, which increases the detection speed by 30%. The efficient detection of aluminum surface defects is realized.

## Introduction

Aluminum is widely used in many fields, such as infrastructure, transportation, aerospace, and industrial manufacturing^1^. In the industrial aluminum production process, aluminum surface defects will inevitably appear, and these aluminum surface defects will seriously affect aluminum quality and performance. Therefore, accurate and fast automatic identification of aluminum surfaces is necessary.

Recently, with the development of machine learning, many scholars have applied this technology to detecting industrial metal surface defects. Wei et al.^2^ proposed a multiscale defect detection network based on a faster R-CNN. The author added the idea of feature fusion to the faster R-CNN to improve detection performance. The improved multiscale defect detection network on the aluminum dataset achieved a higher mAP of 75.8%. Zhang et al.^3^ used the improved YOLOv3 to detect the surface defects of steel strips, and the detection accuracy was only 82.73%. Liu et al.^4^ proposed a feature enhancement and selection module (FESM) to enhance single-scale features and select multiscale features to reduce confounding information. The mean accuracies of the NEU-DET and GC10-DET datasets were 79.4% and 71.6%, respectively. Although the above-improved method can automatically detect metal surface defects, it is difficult to accurately identify metal surfaces in industrial production.

Zhang et al.^5^ proposed an improved YOLOv3 algorithm to detect aluminum defect datasets, and the average accuracy of the improved algorithm in the aluminum dataset reached 87.1%. Li et al.^6^ proposed an improved YOLOv4 algorithm for defect detection in industrial steel. The author designed a convolutional block attention module (CBAM) for backbone networks and a structure similar to receptive field blocks (RFB) to replace the enhanced path aggregation network (PANet) to enhance the network's information acquisition and feature extraction capabilities. The average accuracy of the proposed algorithm in detecting three types of steel strip defects reached 87.85%. Wang et al.^7^ proposed a complex and efficient target detection model for aluminum surface defect detection based on layered attention and context information. According to the characteristics of aluminum surface defect data, the author used adaptive deformation convolution in the feature extraction stage, which enhanced the adaptability of the network to irregular and large deformations of the target and effectively improved the detection performance of the network. Guan et al.^8^ proposed a steel surface defect recognition algorithm based on an improved deep learning network model based on feature visualization and quality evaluation. The authors verified that the recognition effect of six common steel surface defects achieved good recognition accuracy. The above networks all focus on the detection accuracy of the network, but there is little research on the lightweight nature of the network. In industrial aluminum manufacturing, high-strength manufacturing in the actual production line will lead to defects on the aluminum surface. Although large model architectures can achieve accurate performance, their training time and reasoning speed are slow in the actual situation, so the real-time accuracy of product surface defect detection is particularly important. The lightweight processing of real-time detection models can reduce the proportion of network models and the number of model parameters and improve the execution efficiency of the model.

SqueezeNet^9^ was the first lightweight model designed. It replaces the 3 × 3 convolution kernel with a 1 × 1 convolution to reduce computation. MobileNetV1^10^ replaces the 3 × 3 standard convolution with a depth-separable convolution (DSC) block. MobileNetV2^11^ proposes an inverted residual block based on MobileNetV1. Compared with the standard convolution, it has fewer computations and more flexible methods for raising and lowering dimensions. The MobileNetV3^12^ structure improves the feature extraction capability of the network by adding SE blocks and changing the activation function to h-swish. ShuffleNetV1^13^ introduced channel shuffling to enhance the information interaction between different groups. ShuffleNetV2^14^ solves the problem of long convolution time-consuming graphics processing units (GPUs). Compared to ShuffleNetV1, the group convolution operation is removed, and the add operation is replaced with concat. GhostNet^15^ proposes a simple architectural design that replaces part of the convolution computation with inexpensive linear operations to generate more feature maps.

Relevant scholars carried out lightweight processing in the YOLO series model, realized target area detection, and greatly reduced the number of calculations and model volume. In lightweight network design, References^16–18^ adopted a lightweight network as the new backbone network and replaced standard convolution with deep separable convolution, which reduced the number of parameters in the network but also reduced the target detection accuracy. References^19,20^ introduced additional modules while designing lightweight networks. The addition of additional modules can make up for model accuracy, but it requires more computations and a more complex network structure. In Refs.^21,22^, network pruning, parameter quantization, low-rank decomposition, knowledge distillation, and other common methods are usually adopted for the trained model to reduce the number of parameters and calculations. After the suitable application of such methods, the lightweight network task can usually be effectively completed, but the ideal pruning proportion or stable model can be obtained after several experiments. Additionally, the models obtained by pruning and compression have poor universality for different datasets.

In conclusion, a lightweight aluminum surface defect detection model, M2-BL-YOLOv4, is proposed in this paper. First, the inverse residual structure in the MobileNetV2 network is used to replace the residual structure of the YOLOv4 backbone network, reduce the number of parameters and model size, and improve detection speed. Second, a new feature fusion network, BiFPN-Lite, is designed, which introduces a lower cost calculation to improve the feature information aggregation ability, reduce the impact of a lightweight backbone network on detection accuracy, and realize efficient detection of aluminum surface defects in industrial production.

## Research method

### YOLOv4 network

**Figure 1:**
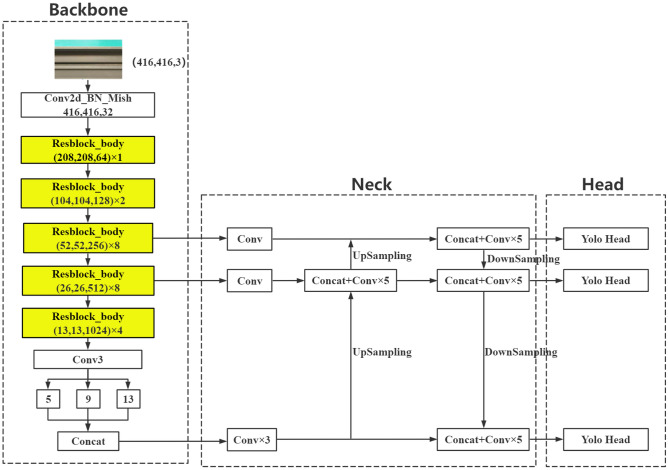
YOLOv4 network structure diagram.

YOLOv4 is a one-stage target detection algorithm with strong real-time performance. The network structure is shown in Fig. 1. The model is divided into four parts: input, backbone, neck, and head. At the input, the picture is uniformly scaled to 416 × 416 size, and mosaic data enhancement adopts random scaling, random cropping, and random arrangement of four pictures for stitching. This greatly enriches the detection dataset, especially since random scaling adds many small targets, making the network more robust.

The backbone network uses CSPDarknet-53 to extract feature information. CSPDarknet-53 introduces the CSPNet structure based on the residual block of Darknet-53, the YOLOv3 backbone network. The CSPNet module first divides the residual blocks into two parts and then combines them through the cross-stage hierarchy to enhance the feature extraction capability of the convolutional neural network, which can improve memory utilization while maintaining accuracy. The neck is divided into spatial pyramid pooling (SPP) and PANet structures. The SPP structure maximizes the bottom feature output of the backbone network by pooling cores of different sizes to enrich the expression ability of the feature graph. The PANet structure is based on the feature pyramid network (FPN) structure to add subsampling, enhance multiscale feature fusion, and realize the full aggregation of strong location information and strong semantic information parameters. Based on the ideas of regression and classification, detection predicts feature maps of three sizes through 3 × 3 and 1 × 1 convolution layers. The dimension of the output result is w × h × 3 × (4 + 1 + num_classes), where num_classes represents the number of categories. YOLOv4 makes predictions for each feature map grid using the prediction information to compare with the real information. The loss function is the evaluation criterion for prediction information and real information. The smaller the loss function is, the closer the prediction information is to the real information. YOLOv4 losses mainly include bbox_loss (bounding box loss), cls_loss (classification loss), and obj_loss (confidence loss).

### Inverted residual block

**Figure 2:**
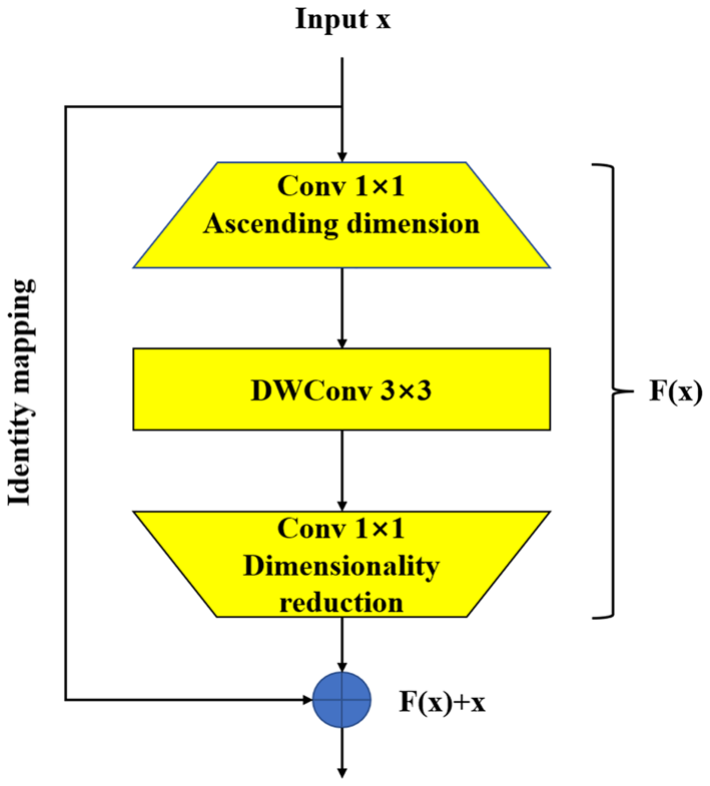
Inverted residual block.

Based on MobileNetV1, the inverted residual structure is proposed in the MobileNetV2 network. The inverted residual structure is the inverted mode of the residual structure. The residual structure has been shown to help improve accuracy by introducing residual edges in the input and output and creating a process of dimensionality first reduction and then expansion; that is, input is first reduced by 1 × 1 convolution pair channels, then 3 × 3 convolution is used to learn the feature information of the target, and finally 1 × 1 convolution pair channel expansion. However, the essential characteristics of the deep convolutional layer cannot change the input feature dimension. If it is compressed first, the feature space will be smaller, resulting in less information that can be extracted. Therefore, the inverted residual block is proposed in the MobileNetV2 network based on the inverted residual structure, that is, the inverted residual structure, as shown in Fig. 2. The process by which dimensions are first expanded and then reduced. The input is amplified by a pointwise convolution operation to enrich the feature space. Then, a 3 × 3 deep convolution is used to extract the feature information, and the activation function is ReLU6. The feature channel is reduced by a pointwise convolution operation, the activation function is linear, and a residual edge is introduced between the input and output to improve accuracy.

### Feature fusion network

**Figure 3:**
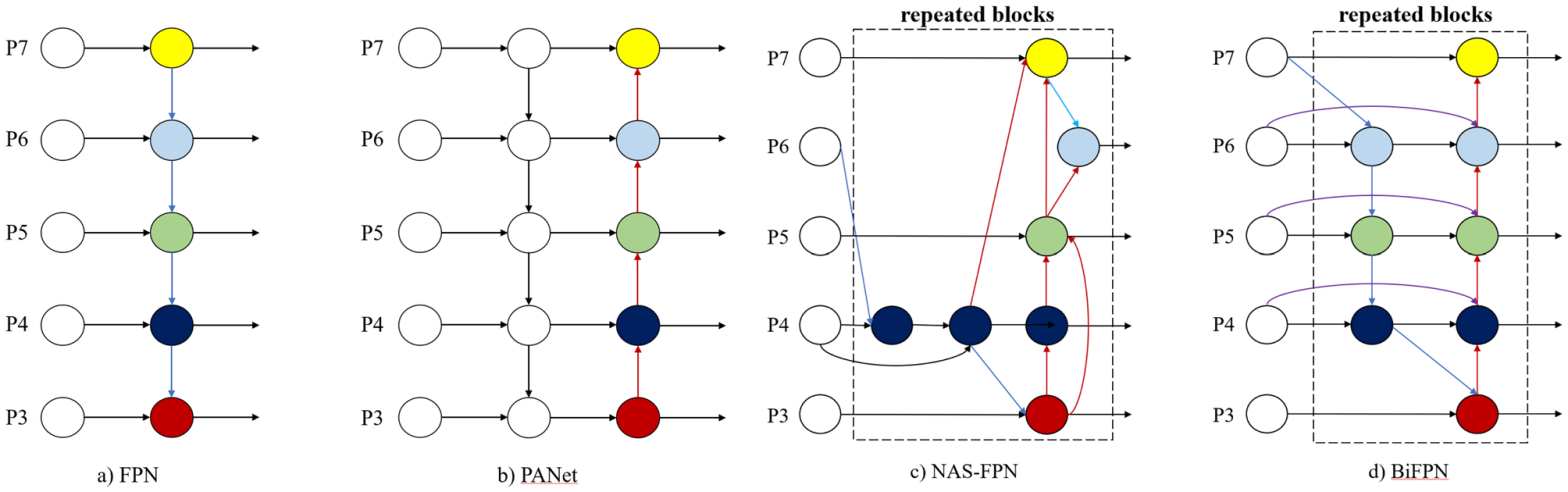
Feature fusion networks.

In the initial network structure, the deep features are extracted from the backbone network for direct prediction, which has no feature fusion, resulting in low detection accuracy. With an in-depth study of the network structure, an FPN network^23^ based on the idea of feature fusion is proposed. The structure is shown in Fig. 3a. A new top-down path for feature fusion can improve prediction accuracy. However, the FPN network has a top-down structure. Due to the authority of single-direction information transmission, it is still difficult to meet the requirements. However, the PANet network with the highest frequency in recent years, among which YOLOv4 uses it as a neck, has a structure as shown in Fig. 3b, which establishes a bottom-up channel on the basis of FPN. The deep feature map has stronger semantic information, which is conducive to object classification, while the shallow feature map has stronger position information, which is conducive to object localization. Such a structure can greatly improve the accuracy of target detection tasks.

Additionally, the recently proposed NAS-FPN structure^24^, the specific structure of which is shown in Fig. 3c below, uses neural architecture search (NAS) technology to search for the best network structure. Although this structure works best, the search-based network is irregular, and the use of NAS technology is time-consuming and work-intensive and is not widely used.

Based on this, a novel neck network structure, BiFPN^25^, is proposed. BiFPN, as a feature fusion network, helps the network obtain a more advanced feature fusion mode, increases feature fusion at different scales, and introduces a weighted feature fusion mechanism, as shown in Fig. 3d below.

Fast normalized fusion is used to learn the weights in the weighted feature fusion mechanism, which is faster and more efficient than other methods. As shown in Eq. (1), where i = j is the number of fused feature graphs at the node, the initial value of the weight is randomly selected between 0 and 1, which is used to make the nonzero constant denominator, wi is ensured by adding ReLU after each wi >  = 0, and e = 0.0001 is a small value to avoid numerical instability. This method reduces the weight range to [0, 1] and obtains the optimal weight after multiple training sessions. The importance of each input is represented in the fusion node.

1 $$\begin{array}{c}Output=\sum_{\mathrm{i}}^{ }\frac{{\upomega }_{\mathrm{i}}^{ }}{\upvarepsilon +\sum_{\mathrm{j}}{\upomega }_{\mathrm{j}}}\cdot {\mathrm{I}}^{\mathrm{i}}\end{array}.$$

## Lightweight network design

### Backbone network lightweight

To enable the CNN model to learn more abundant feature information, many researchers have continuously stacked convolution layers and pooling layers from the perspective of increasing the depth of the network. For example, ResNet series models have extended the number of model layers from the initial 18 layers to 50 layers, 101 layers, and even 152 layers by using the residual connection method. Although the design method of stacking layers can improve the performance of the model, the number of parameters and calculation cost of the model are constantly rising, and the cost performance curve composed of the number of network layers and accuracy is gradually becoming saturated. The main idea of lightweight model design in this paper is to redesign the YOLOv4 network. For the backbone network, a lightweight model is adopted to replace a large number of stacked residual structures, and the MobileNetV2 network is taken as the backbone extraction network of YOLOv4 to form the M2-YOLOv4 network model. The number of parameters and computations of the backbone network are reduced to reduce the storage volume of the entire network model and improve the running speed of the model. Table 1 shows the detailed composition of the M2-YOLOv4 backbone network.

**Table 1:** The M2-YOLOv4 backbone network composition.

Operator	Channel	Number	Stride	Output
Input	3	–	–	416 × 416 × 3
Conv2d_BN_ReLU6	32	1	2	208 × 208 × 32
InvertedResidual_block	16	1	1	208 × 208 × 16
InvertedResidual_block	24	2	2	104 × 104 × 24
InvertedResidual_block	32	3	2	52 × 52 × 32
InvertedResidual_block	64	4	1	26 × 26 × 64
InvertedResidual_block	96	3	1	26 × 26 × 96
InvertedResidual_block	160	3	2	13 × 13 × 160
InvertedResidual_block	320	1	1	13 × 13 × 320

**Figure 4:**
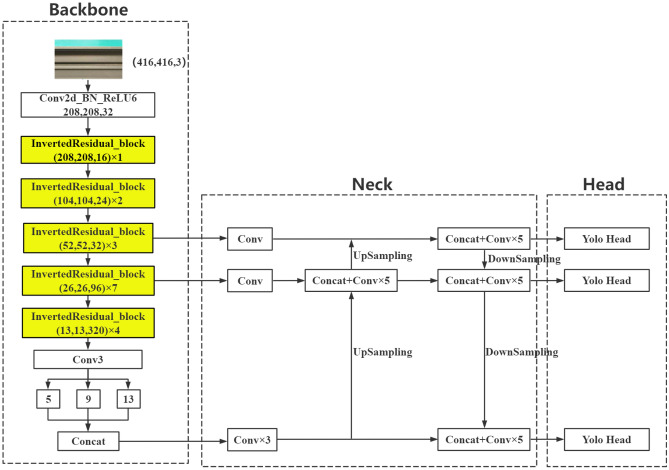
The M2-YOLOv4 network structure diagram.

By inputting 416 × 416 images and outputting 208 × 208 feature images with 32 channels through convolution, the number of channels input from the previous layer is halved, and five inverted residual structures are stacked 1, 2, 3, 7, and 4 times to form a feature extraction network. The image input of 416 × 416 × 3 is extracted through the backbone features to extract the feature map of 13 × 13 × 320 for the neck network to fuse. M2-YOLOv4 mainly uses the inverted residual module in MobileNetV2. The inverted residuals module first expands the input features to a higher dimension by a 1 × 1 convolution, then performs a 3 × 3 depth-separable convolution, and finally uses a 1 × 1 convolution for dimensionality reduction. The inverted residuals module uses jump joins if and only if the input and output have the same number of channels. The inverted residual module extends internally into high-dimensional space to improve the representation of nonlinear all-channel transformations while maintaining a compact representation of inputs and outputs. Figure 4 shows the complete network structure of M2-YOLOv4.

### Improved feature fusion

After the lightweight model is adopted in the backbone network, the network parameters can be reduced, the detection speed can be increased, and the acquisition of target feature information can be weakened to a certain extent. Therefore, to reduce the impact on the detection accuracy, the feature fusion part of the neck is proposed to be improved to achieve efficient aggregation of multiscale features.

In the introduction of the feature fusion network, the standard BiFPN structure downsamples the original image to obtain five scales for feature fusion, while the YOLOv4 network adopts the backbone extraction network to output three scale feature layers of 13 × 13, 26 × 26 and 52 × 52 for feature fusion, and the feature scale of the bottom layer is 13 × 13. It is difficult to downsample again. Therefore, when combined with the YOLOv4 network, two feature layers of the BiFPN structure are removed so that the BiFPN structure matches the PANet structure in YOLOv4, feature fusion is only realized for three scales, and a residual edge from input to output is added in the middle layer P4. Additionally, the weighted feature fusion mechanism is introduced in the nodes where P4_td, P3_out, P4_out, and P5_out have multiple inputs. The improved BiFPN structure is called the BiFPN-Lite structure.

BiFPN-Lite features a fusion mechanism with weights, generates weights with different contribution degrees of multiple input features by introducing a simple attention mechanism idea and learns these weights using a fast normalization method. We multiply the feature mapping and weight at the feature fusion node to focus the network on the more important features. P4_td and P4_out nodes are taken as examples to describe the weighted fusion properties. Equations (2) and (3) are obtained from Eq. (1), where Conv5 represents the convolution operation. Resize denotes upsampling or downsampling operations and represents the weight corresponding to each input feature map. ε is a constant that causes the denominator to be nonzero.

2 $$\begin{array}{*{20}c} {{\text{P}}_{{4_{{{\text{td}}}} }} = Conv5\,\left( {\frac{{\omega_{1} \, \cdot {\text{P}}_{{4_{{{\text{in}}}} }} + \omega_{2} \cdot {\text{Resize}}\,\left( {{\text{P}}_{{5_{{{\text{in}}}} }} } \right)}}{{\omega_{1} + \omega_{2} + \varepsilon }}} \right)} \\ \end{array},$$

3 $$\begin{array}{*{20}c} {{\text{P}}_{{4_{{{\text{out}}}} }} = Conv5\,\left( {\frac{{\omega_{1} \cdot {\text{P}}_{{4_{{{\text{in}}}} }} + \omega_{2}{\prime} {\text{P}}_{{4_{{{\text{td}}}} }}^{ } + \omega_{3}{\prime} \cdot {\text{Resize}}\left( {{\text{P}}_{{3_{{{\text{out}}}} }} } \right)}}{{\omega_{1}{\prime} + \omega_{2}{\prime} + \omega_{3}{\prime} + \varepsilon }}} \right)} \\ \end{array} .$$

**Figure 5:**
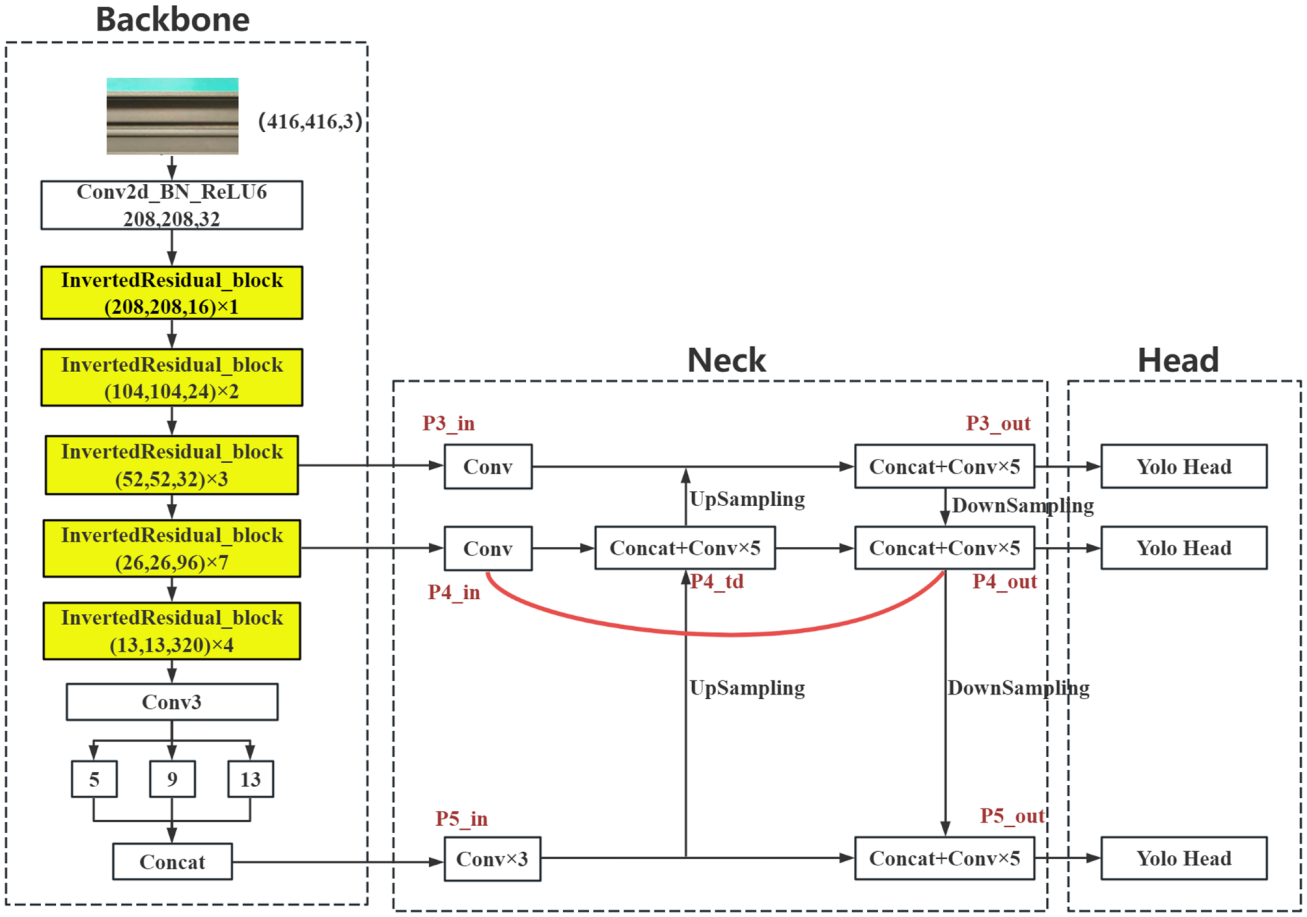
The M2-BL-YOLOv4 network structure diagram.

The lightweight network model based on the improved BiFPN is called M2-BL-YOLOv4, and its structure is shown in Fig. 5. P5_in is directly upsampled, and P4_in is connected at the P4_td node. P4_td upsampling is connected with P3_in to output P3_out. The results of P4_in, P4_td, and P3_out downsampled are connected to output P4_out, and the connection between P4_in and P4_out forms a residual edge. P5_in and P4_out downsampling results are connected to output P5_out. After the outputs P4_out, P3_out, and P5_out of the BiFPN-Lite feature fusion structure are sent to the detection layer, the YOLO Head predicts the result of the feature map of the three sizes.

## Experimental process and results

### Data sample introduction

**Figure 6:**
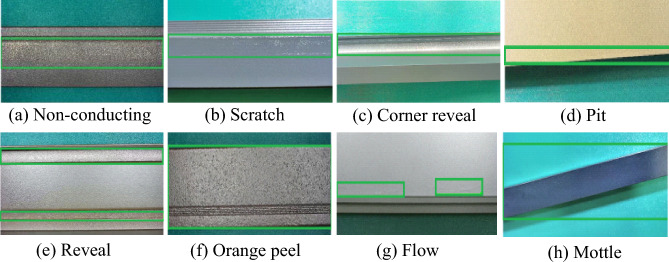
Eight types of defects on the aluminum surface.

The experimental dataset for aluminum surface defect detection is from the target detection competition of the Guangdong Industrial Manufacturing Big Data Innovation Competition. The resolution of the images for machine learning is 2560 × 1960, and the selected defects include non-conducting, reveal, scratch, orange peel, corner reveal, flow, pit, and mottle in a total of eight categories. LabelImg is used to label single-target defects or multitarget defect images. The underlying aluminum surface defect data sample is small, with only 2400 defect images in the original dataset, which can lead to overfitting problems during training. Therefore, to improve matching the dataset with the complex model and enhance the generalization ability of the algorithm, data enhancement is performed on the existing defect samples. First, the aluminum dataset was divided into training and test sets at a ratio of 85:15, and then the data were expanded in the training and test sets using three methods: horizontal, vertical, and horizontal-vertical. The total number of data samples obtained after enhancement is 9600, of which the number of the training set is 8160 and the number of the test set is 1440. The data format of this dataset is in the form of VOC, and the experiments in this paper are based on this dataset. The eight types of defects in the aluminum dataset are shown in Fig. 6.

### Experimental environment and model training

In the experimental environment of the Windows 10 system and NVIDIA and CUDA-driven graphics cards, Pytorch is chosen as the deep learning framework. Compared with other common frameworks, PyTorch is a widely used research tool in the direction of machine learning and deep learning with high flexibility, ease of use and fast running speed.

In this experiment, the pretraining weight of VOC data was used to improve the training efficiency of the network. There were 300 epochs in the whole training process, and the weight attenuation coefficient was 0.0005. The freezing training mode divided the whole training into two stages, with the learning rate of the first half being 8 and the batch of input images being 4. The freezing training mode can improve the convergence speed of the network and prevent the network weight from being destroyed in the early stage of training.

### Description of evaluation index

The intersection ratio between the predicted box and the real box is set to the IOU threshold of 0.5. If the IOU is greater than 0.5, the target is successfully detected. AP represents the average accuracy of a certain class. In Eq. (4), P represents the accuracy rate, R represents the recall rate, P is the functional relationship with R as the variable, and the integration of the P(R) function is defined as the value of AP. The mAP is the average statistic of average accuracy, and in Eq. (5), K is the total number of categories and the average of the AP values of all categories. The change in mAP can more intuitively show the accuracy of the model detection. The larger the mAP value is, the higher the detection accuracy.

4 $$\begin{array}{c}AP={\int }_{ }^{ }\mathrm{P}\left(\mathrm{R}\right)d\mathrm{R}\end{array}$$

5 $$\begin{array}{c}mAP=\frac{\sum \mathrm{AP}}{\mathrm{K}}\end{array}$$

The detection time was evaluated using image detection and the frame rate FPS. As shown in Eq. (6), the average image detection time is the total detection time divided by the number of detection times. As shown by Eq. (7), the frame rate is FPS. The shorter the time spent on a single image, the larger the FPS result, indicating that the detection speed of the network model is faster.

6 $$\begin{array}{c}Time=\frac{\mathrm{Total \,Time}}{\mathrm{Test\, Interval}}\end{array}$$

7 $$\begin{array}{c}FPS=\frac{1}{\mathrm{Time}}\end{array}$$

### Result analysis

**Figure 7:**
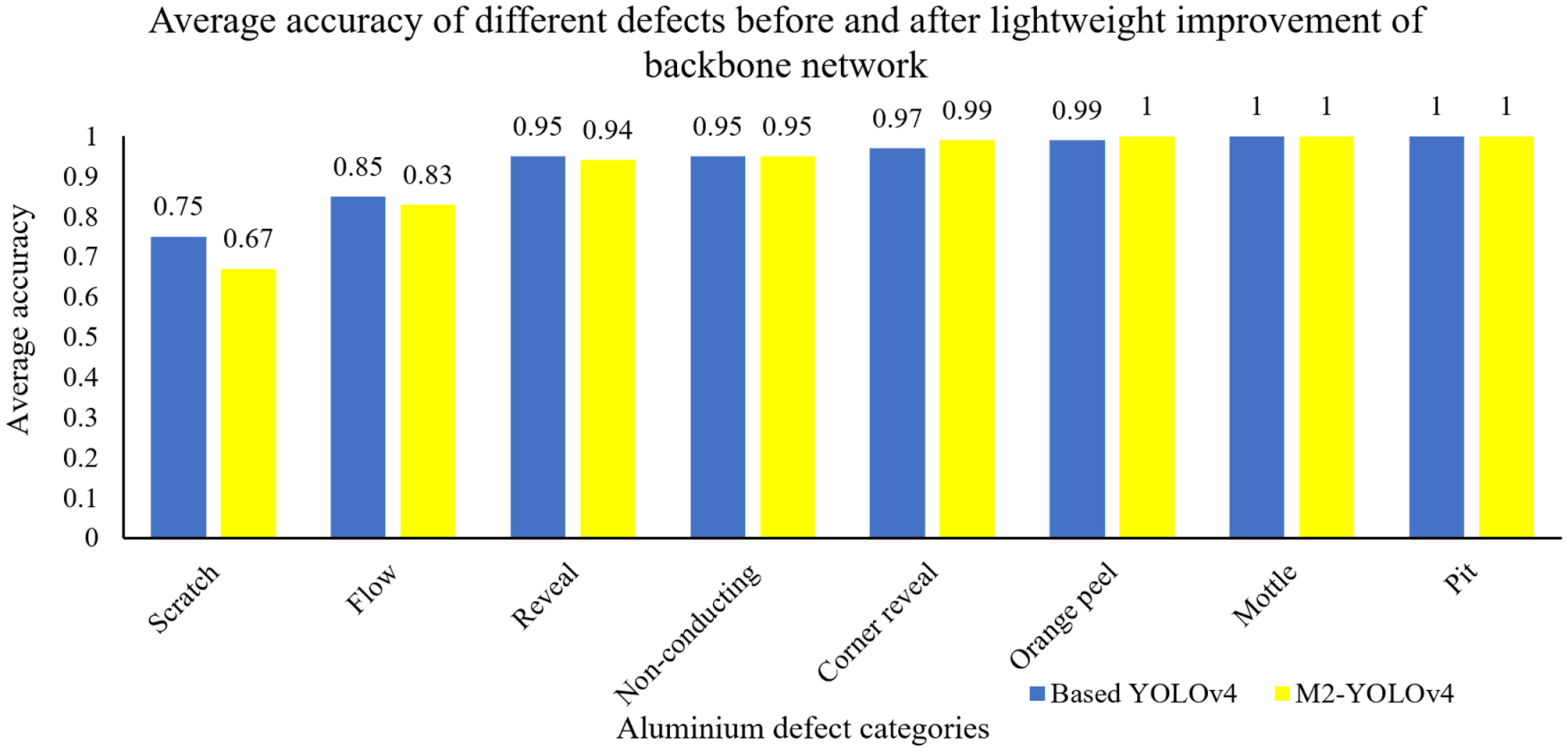
The AP value of defects before and after lightweighting in the backbone.

The backbone network of YOLOv4 improved from CSPDarkNet53 to MobileNetV2, and the lightweight network model of M2-YOLOv4 was formed. The operation method based on depth-separable convolution greatly reduces the number of parameters and calculations. To study the impact of network light weighting improvement on the detection performance of aluminum surface defects, 8160 images were trained, 1440 images were detected, and the ratio of training and testing on the dataset was 85:15. The predicted results were compared with the basic YOLOv4 network. Table 2 shows the comparison of the model detection performance before and after the network lightweight improvement, and Fig. 7 shows the comparison of the average accuracy of the model on various defects.

**Table 2:** Detection performance before and after lightweighting in the backbone.

Network model	mAP@0.5	Time	FPS
Based YOLOv4	93.31%	0.0251 s	39.80
M2-YOLOv4	92.25%	0.0190 s	52.79

As shown in Table 2, the mAP of the basic YOLOv4 algorithm is 93.31%, and after the backbone extraction network is lightened, the mAP is reduced to 92.25%. The detection accuracy of all kinds of defects is shown in Fig. 7. Compared with the basic network, the average accuracy value of the defects of the non-conducting, reveal, flow, and scratch has a poor recognition effect because the lightweight structure effectively reduces the model calculation intensity and the complexity of the network, affecting the feature extraction and resulting in greater accuracy loss. However, in terms of detection time, it is found that the lightweight model reduces the parameter size and memory consumption, the detection time is reduced from 0.0251 s to 0.0190 s, the FPS is increased by approximately 33%, and the detection speed is greatly improved.

When MobileNetV2 is selected as the lightweight backbone network, a comparative experiment is carried out. One is YOLOV4-Tiny, a lightweight network based on YOLOv4. The other is for the backbone network of YOLOv4, MobileNetV1, MobileNetV2, MobileNetV3, and GhostNet are used as the backbone extraction network of YOLOv4. The network models of M1-YOLOv4, M2-YOLOv4, M3-YOLOv4, and G-YOLOv4 were formed. We train on the same dataset and obtain the prediction results on the same test set. The comparison of the parameters of each model is shown in Table 3.

**Table 3:** Comparison of the parameters of each model.

Network model	Parameter	Model size (MB)
YOLOv4	64,040,001	244.29
M1-YOLOv4	40,952,893	156.22
M2-YOLOv4	39,062,013	149.01
M3-YOLOv4	39,989,933	152.55
G-YOLOv4	39,689,409	151.40
YOLOv4-tiny	5961,014	22.74

As seen in the comparison of the parameters of each model in Table 3, YOLOv4 has the largest number of basic network parameters, and the model size is 244.29 MB. YOLOv4-tiny Perform compression based on YOLOv4 with the smallest number of parameters. The network model formed by the three backbone networks replaced by CSPDarknet and MobileNet is reduced to 63.9%, 60.9%, and 62.4%, respectively, and the network model replaced by GhostNet is reduced to 61.9%. Therefore, replacing the original backbone network of YOLOv4 with the lightweight network can greatly reduce the model parameters and size. The comparison of the detection accuracy of each model is shown in Table 4, where P denotes pit, M denotes mottle, Op denotes orange peel, Cr denotes corner reveal, Nc denotes non-conducting, R denotes reveal, F denotes flow, and sc denotes scratches.

**Table 4:** Comparison of the detection precision of each model.

Network model	mAP@0.5	P	M	Op	Cr	Nc	R	F	S
YOLOv4	93.31%	1	1	0.99	0.97	0.95	0.95	0.85	0.75
M1-YOLOv4	92%	1	1	1	0.99	0.95	0.95	0.78	0.69
M2-YOLOv4	92.25%	1	1	1	0.99	0.95	0.94	0.83	0.67
M3-YOLOv4	91.13%	1	1	1	0.99	0.95	0.94	0.73	0.68
YOLOv4-tiny	82.25%	0.95	1	0.94	0.97	0.86	0.86	0.54	0.46
G-YOLOv4	88.5%	0.99	1	1	0.99	0.95	0.95	0.72	0.48

We can see this in Tables 3 and 4. In YOLOV4, the CSPDarkNet53 network was used to extract feature information, and the feature information was aggregated through the SPP and PANet structures to detect more complex features. However, deepening or broadening the network resulted in more parameters and larger models. The mAP of YOLOv4-tiny is only 82.25%, which is because the network structure is too simple. The feature information is seriously lost, and the detection accuracy is seriously affected, especially the complex and small defects that cannot extract significant and rich feature information, such as flow(F) and Scratch(S). Therefore, compared with other models, the detection accuracy is quite different. Ghost-YOLOv4 has better detection performance than YOLOv4-tiny but worse performance than the MobileNet series. The detection effects of MobileNetV1 and MobileNetV2 are similar, and the defect detection accuracy of MobileNetV2 is higher than that of MobileNetV3. Therefore, the model parameters can be reduced to a certain extent when the backbone network is improved to meet the requirements of lightweight networks for the detection accuracy of aluminum surface defects. Through experimental comparison, MobileNetV2 was selected to replace CSPDarkNet53 as the improved YOLOv4 backbone extraction network by reference to the detection accuracy in the above network model, the final network model was formed as M2-YOLOv4, and then the lightweight network was further optimized.

When the backbone network is lightweight, the feature information extracted from the backbone network is limited, resulting in low target detection results. Therefore, the BiFPN-Lite structure is proposed to achieve efficient aggregation of the backbone network's output multiscale feature information to improve detection accuracy. To verify the validity of the BiFPN-Lite structure, comparative tests were conducted using different feature fusion networks with the same dataset. The experimental results are shown in Table 5.

**Table 5:** Parameters and detection performance of different feature fusion models.

Network model	mAP@0.5	Time	FPS	Parameter
M2 -FPN-YOLOv4	92.1%	0.0183 s	53.17	37,541,741
M2 -PANet-YOLOv4	92.25%	0.0190 s	52.79	39,062,013
M2-NASFPN-YOLOv4	93.26%	0.0231 s	50.26	42,753,128
M2-BL-YOLOv4	93.5%	0.0188 s	52.99	39,234,339

As shown in Table 5, although the M2-FFPN-YOLOV4 network structure has the fastest reasoning speed and the smallest number of parameters for aluminum surface defect detection, the mAP of the M2-BL-YOLOv4 network is 1.4% higher than that of the M2-FFPN-YOLOV4 network structure. In industrial metal surface defect detection, the task must have rapid detection ability, but excellent detection performance is more important. The M2-NASFPN-YOLOv4 network also has excellent detection ability, but the number of references is 8.9% higher than the M2-BL-YOLOv4 network, and the reasoning speed is also slower than the improved network. When PANet was modified into a BiFPN-Lite structure, the number of parameters in the M2-BL-YOLOv4 network only changed slightly compared with the M2-PANet-YOLOv4 network, and the number of parameters was reduced by 39% compared with the Based YOLOv4 network. In terms of detection accuracy, mAP reached 93.5%. The improved detection accuracy is slightly higher than the base value. Table 6 shows the comparative analysis of the detection accuracy of the improved network, other classical networks, and networks in other literature for various defects in aluminum datasets.

**Table 6:** Comparative experiments on improving networks and other networks on aluminum datasets.

Network model	mAP@0.5	P	M	Op	Cr	Nc	R	F	S
YOLOv4	93.31	1	1	0.99	0.97	0.95	0.95	0.85	0.75
M2-YOLOv4	92.25	1	1	1	0.99	0.95	0.94	0.83	0.67
YOLOv5	89.33	0.98	0.98	0.84	0.98	0.86	0.97	0.69	0.82
YOLOv7	92.25	1	0.98	0.86	1	0.9	0.97	0.86	0.81
Li’s method^26^	87.38	1	0.98	0.98	1	0.83	0.93	0.73	0.54
Hao’s method^27^	90.75	1	0.97	0.97	0.92	0.92	0.91	0.89	0.68
M2-BL-YOLOv4	93.5	1	1	1	0.99	0.96	0.95	0.85	0.73

**Figure 8:**
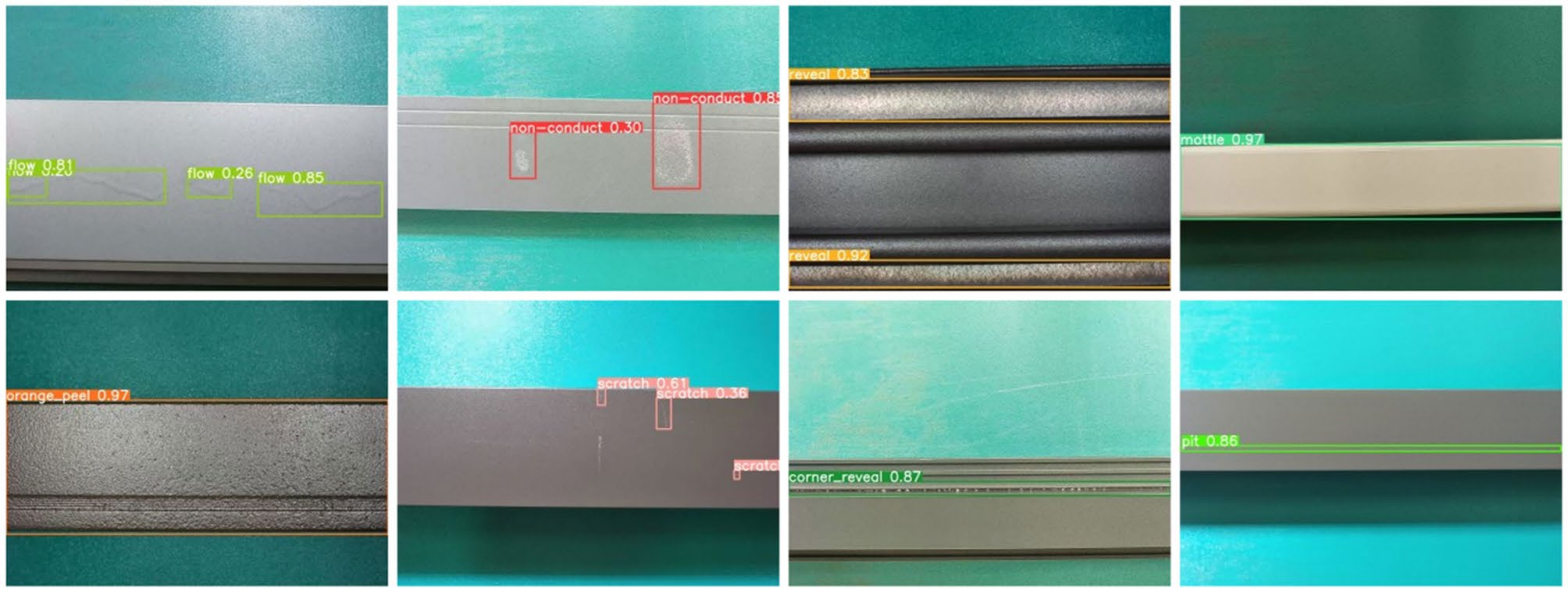
Detection results of the M2-BL-YOLOv4 model.

The average detection accuracy of various defects is shown in Table 6. When compared with the detection accuracy based on YOLOv4, M2-BL-YOLOv4 improved the orange peel and corner reveal defect type detection. When compared with M2-YOLOv4, M2-BL-YOLOv4 improves the detection accuracy of non-conducting and reveal. In mottle and orange peel defect detection, M2-BL-YOLOv4 has the best detection effect, and among the compared methods, M2-BL-YOLOv4 has the best detection performance, with an mAP of 93.5%. Experimental results show that in the fusion of BiFPN-Lite and YOLOv4, the residual edges of the input and output of the P4 layer are introduced into the network structure, and the importance of learning different input features in the feature fusion node is enhanced, which enhances the degree of feature aggregation and improves the detection accuracy of the model without increasing the cost too much. The M2-BL-YOLOv4 model still maintains advantages in detection time on the premise of ensuring detection accuracy and enhancing the ability of network real-time detection. Figure 8 shows some test results of our proposed M2-BL-YOLOv4 model.

### Related experiments on NEU-DET datasets

To further explore the validity and generalization of our proposed method, we conducted experiments on the NEU-DET dataset. As shown in Table 7, cr denotes crazing, in denotes inclusion, pa denotes patches, ps denotes pitted surface, rs denotes rolled-in scale, and sc denotes scratches.

**Table 7:** Experimental results on the NEU-DET dataset.

Network model	mAP@0.5	cr	in	pa	ps	rs	sc
YOLOv5s	82.8	60.8	62.7	83.8	92.9	63.6	69.4
YOLOv4	82.3	66.8	56.6	87.4	96.5	57.3	54.5
YOLOv7	74.9	76.3	63.4	81.3	78.3	82.4	95.5
PPYOLO	79.7	55.1	51.9	77.3	87.6	49.5	58
Zhao’s method^28^	81.1	52.9	85.9	94.4	86.2	70.7	96.6
Yu’s method^29^	72.8	15.1	26.3	42.4	33.8	27.2	30.6
M2-BL-YOLOv4	84.3	75.5	72.8	87.2	97.0	79.9	93.4

**Figure 9:**
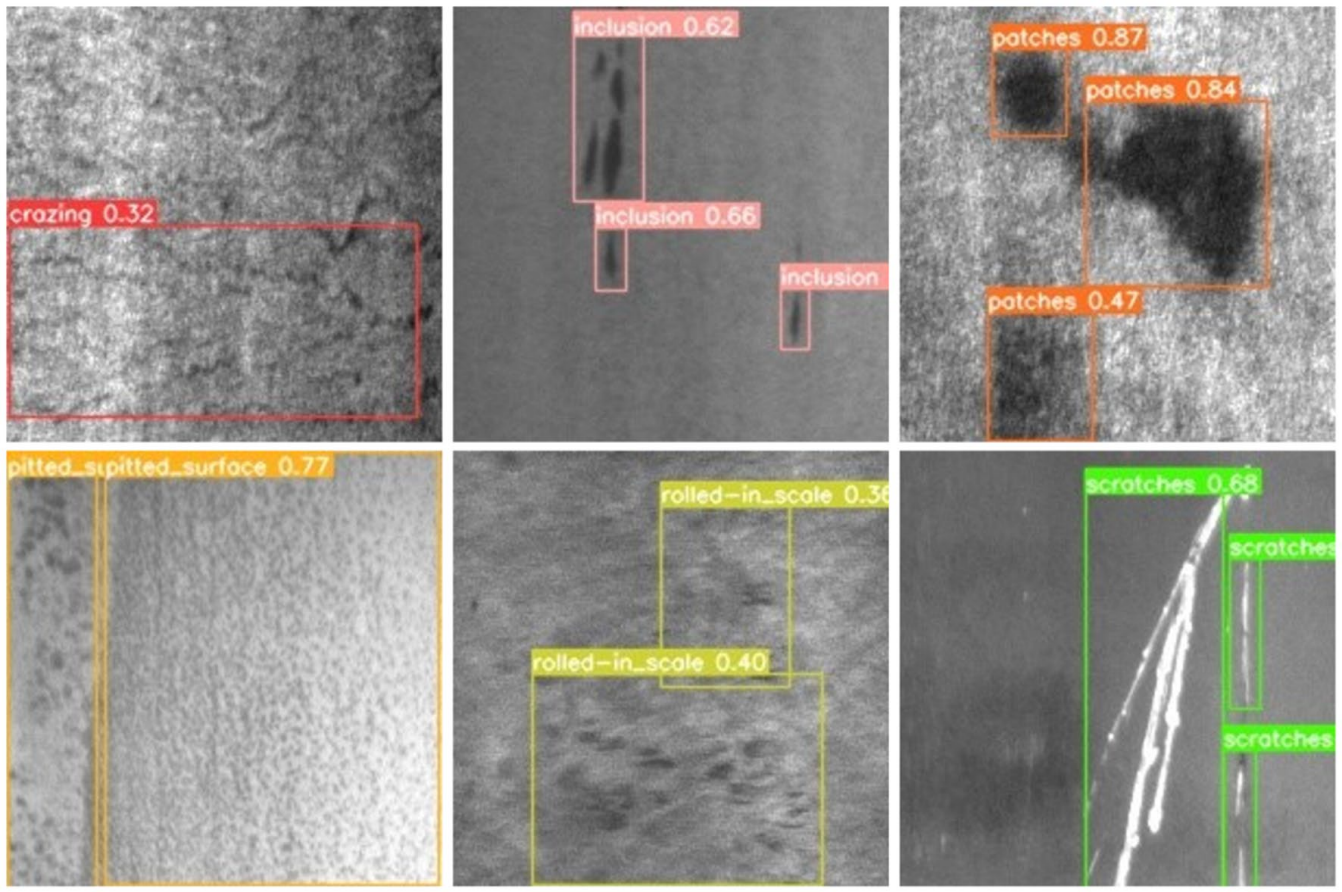
Detection results of the M2-BL-YOLOv4 model on the NEU-DET dataset.

We compare it with several mainstream methods. Table 7 shows that the highest mAP is 84.3%, which is achieved by M2-BL-YOLOv4. The mAP of our proposed model increases by 1.5% compared with YOLOv5s, and it performs best in the pitted surface (ps) defect category. The mAP of M2-BL-YOLOv4 is 9.4% higher than that of YOLOv7, and although Zhao’s method^28^ achieves the highest accuracy in the inclusion (in), patches (pa) and scratches (sc) defect categories, the mAP is 3.2% lower than that of our proposed model. Compared with Yu^29^, the mAP of M2-BL-YOLOv4 is 11.5% higher than that of Yu. Experimental results show that the M2-BL-YOLOv4 algorithm proposed in this paper has excellent generalization ability and is superior to similar algorithms. Figure 9 shows some detection results of the M2-BL-YOLOv4 detection model on the NEU-DET dataset.

## Conclusion

In this paper, a lightweight aluminum surface defect detection model, M2-BL-YOLOv4, is proposed. First, through the construction of a lightweight model, the MobileNetV2 lightweight network is selected as the backbone feature extraction network to form the M2-YOLOv4 network model. The depth-separable convolution significantly reduces the model size. On this basis, the BiFPN_Lite structure is proposed as the feature fusion network. The BiFPN_Lite structure is an improvement of the BiFPN structure, and the combination of weighted fusion mechanisms can improve the accuracy of the algorithm for defect detection. The results show that the average accuracy of the improved M2-BL-YOLOv4 reaches 93.5%, which is slightly higher than that of the basic network detection, and the number of model parameters is reduced to 60% of the original, greatly reducing the size of the model, and the detection speed FPS is increased from 39.8 to 52.99, which realizes the efficient detection of aluminum surface defects and meets the real-time needs. We also conducted experiments on the NEU-DET dataset. Compared with several mainstream models and methods in other literature, the mAP of M2-BL-YOLOv4 reached 84.3%, which was superior to other detection methods and verified the excellent generalization ability of the proposed method.

## Data Availability

The datasets used and analyzed during the current study are available from the corresponding author upon request.
